# Salivary clearance of caffeine in children

**DOI:** 10.1016/j.jobcr.2023.03.008

**Published:** 2023-03-15

**Authors:** Basant K. Puri, Christopher R. Heard, Jean A. Monro

**Affiliations:** a C.A.R, Cambridge, UK; b University of Winchester, Winchester, UK; c London, UK; d Breakspear Medical Group, Hemel Hempstead, UK

**Keywords:** caffeine, 1,3,7-trimethylpurine-2,6-dione, Salivary clearance, Children, CYP, cytochrome P450

## Abstract

**Background:**

Measurement of salivary caffeine (1,3,7-trimethylpurine-2,6-dione or 1,3,7-trimethylxanthine) clearance can, in principle, be used to assess hepatic function, diagnose chronic hepatic disease and conduct investigations of substrates of hepatic cytochrome P450 (CYP) isozymes in children, without recourse to venepuncture. However, little is known about childhood sexual dimorphism of hepatic CYP isoforms. Furthermore, the association, if any, between salivary caffeine clearance and age in children has not hitherto been established. The aims of this study were to assess whether salivary caffeine clearance differs between boys and girls and whether it varies with age during childhood.

**Methods:**

Following at least 24 h’ abstinence from dietary caffeine, nine boys (mean (standard error) age 9.6 (1.1) y) and eight girls (mean age 11.0 (1.2) y), none of whom was a smoker or suffered from chronic hepatic disease, ingested an oral caffeine dose titrated by body mass, namely 3 mg kg^−1^. Salivary samples collected two and 14 h later underwent spectrophotometric caffeine analysis.

**Results:**

The boys and the girls were age matched. The mean caffeine clearance in the boys was 2.47 (0.33) mL min^−1^ kg^−1^, while that in the girls was 2.20 (0.31) mL min^−1^ kg^−1^ (*p* = 0.56). The salivary caffeine clearance was negatively correlated with age (*r* = −0.59, *p* = 0.01).

**Conclusion:**

Stratification by sex appears to be unnecessary when considering childhood salivary caffeine clearance or when conducting investigations in children of CYP1A2 and xanthine oxidase substrates. Furthermore, childhood salivary caffeine clearance is negatively correlated with age.

## Graphical abstract

**Figure:**
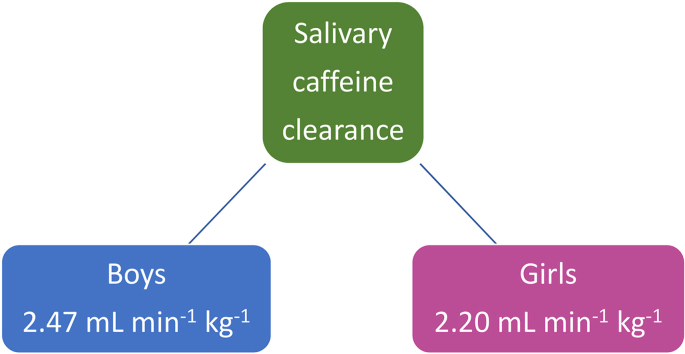

## 1 Introduction

The natural alkaloid most widely consumed by adult humans is 1,3,7-trimethylpurine-2,6-dione, also known as 1,3,7-trimethylxanthine and more commonly as caffeine.^1^ Consumption of up to 400 mg caffeine daily appears to be relatively safe in non-pregnant adults, while the corresponding figure for pregnant women appears to be 300 mg daily.^2^ As might be expected, relatively few exposure-response studies have been carried out in children and adolescents; the consensus safety limit for children is 2.5 mg kg^−1^ day^−1^.^2^^,^^3^ These safety data are potentially useful clinically, as human salivary caffeine clearance can be used as a non-invasive test of hepatic function.^4^ Such non-invasive testing is clearly preferable to venepuncture in children and has been employed in a small number of studies.^5^^,^^6^

In a study of children suffering from cystic fibrosis, the salivary caffeine clearance was found to be lower in seven hepatopathic children compared with non-hepatopathic children; the potential clinical importance of salivary caffeine clearance assessments in such children is evident, given that hepatopathy is a known complication of cystic fibrosis.^7^ Another study of nine paediatric patients with hepatocellular disease also reported lower salivary caffeine clearance compared with nine healthy children.^8^

Prepubertal children with somatotropin deficiency may be offered treatment with recombinant human growth hormone replacement. However, such treatment is reported to be associated with reduced clearance of drugs catabolised by hepatic cytochrome P450 (CYP) enzymes.^9^, ^10^, ^11^ Given the important role of CYP-dependent metabolic pathways in the catabolism of caffeine,^12^ assessment of such children by the salivary caffeine clearance may be of clinical value. Indeed, significant decreased CYP-dependent 3-*N*-demethylation of caffeine, indexed by the ^13^CO_2_-caffeine breath test, has been reported in a cohort of six somatotropin-deficient children after just one month of treatment with growth hormone replacement.^13^

Whilst sexual dimorphism of hepatic isoforms of CYP has been established in species ranging from rats to pigs and mice, and in non-mammalian species such as the chicken and the trout, little is known about sex differences in human childhood and adolescent CYP isoforms.^14^, ^15^, ^16^, ^17^ In 2020, we published a study in this journal showing that there was no sex difference in salivary clearance of caffeine.^12^ A recent perusal of the data has revealed that, of the 213 original subjects, 17 were under the age of 16 years at the time that the caffeine clearance was studied. As nine of these subjects were boys and eight girls, this has afforded us the opportunity to determine whether there was a sex difference in caffeine clearance in children as well as to examine any relationship between caffeine clearance and age. Here, we report the results of our further analysis of these data.

## 2 Materials and method

### 2.1 Subjects

The mean (standard error) age of the nine boys was 9.6 (1.1) years, while that of the eight girls was 11.0 (1.2) years. None of the 17 subjects suffered from chronic liver disease and none was a smoker. The audit was carried out with research ethics committee approval and was carried out in accordance with the Declaration of Helsinki.

### 2.2 Samples

Following at least 24 h’ abstinence from dietary caffeine, each child ingested a single oral caffeine dose, of 3 mg kg^−1^ (body mass), in the morning. Collection of salivary samples took place 2 h and 14 h later. These underwent spectrophotometric caffeine analysis by MetaMetrix Inc. (Norcross, GA).

### 2.3 Statistical analysis

Data analysis was carried out using JASP 0.16.3 and R version 4.2.1.^18^^,^^19^ Group comparisons were carried out using the Student *t*-test, after checking for both normality with the Shapiro-Wilk test and equality of variances using the Levene test. Correlation was tested using the Pearson product-moment correlation coefficient after checking for multivariate normality using the Shapiro-Wilk test.

## 3 Results

There was no significant difference in mean age between the boys and the girls (*t* = 0.88, *df* = 15, NS). The mean caffeine clearance in the boys was 2.47 (0.33) mL min^−1^ kg^−1^, while that in the girls was 2.20 (0.31) mL min^−1^ kg^−1^ (*t* = 0.59, *df* = 15, NS).

**Fig. 1:**
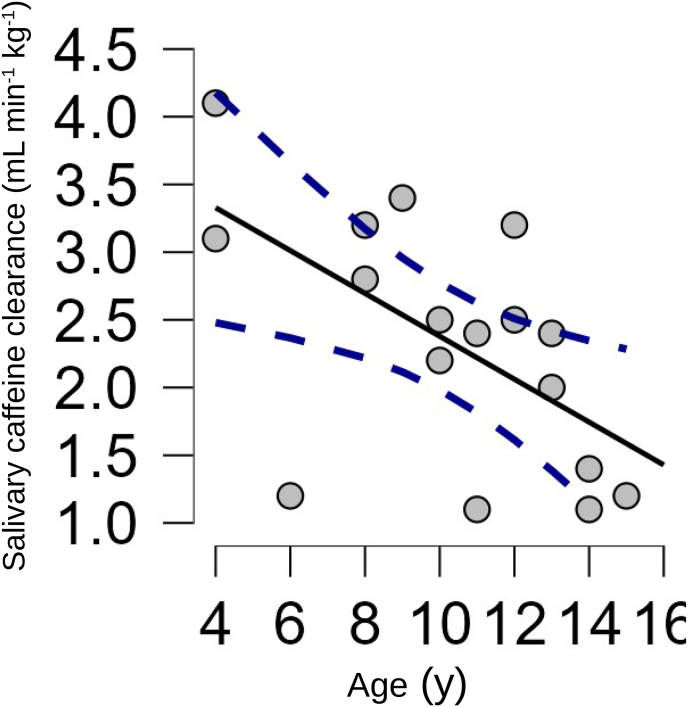
Scatterplot of salivary caffeine clearance versus age. The best-fit linear regression line is shown (continuous line). Its 95% confidence interval is represented by the two dashed lines.

The salivary caffeine clearance was negatively correlated with age (*r* = −0.59, *p* = 0.01). A correlation plot is shown in Fig. 1, which also includes the best-fit linear regression line and its associated 95% confidence interval.

## 4 Discussion

Our data point to two main results. First, there is no evidence of a sex difference in salivary caffeine clearance in children. Thus, there appears to be no requirement for stratification by sex when considering childhood salivary caffeine clearance results and when conducting clinical or pharmacokinetic investigations in children of CYP1A2 and xanthine oxidase substrates. This mirrors a similar conclusion for adult humans by Kashuba and colleagues, on the basis of their study of urinary caffeine metabolite ratios in 10 pre-menopausal women and 10 men.^20^

The second main result is that childhood salivary caffeine clearance is negatively correlated with age. The best-fit linear regression equation is:

Salivary caffeine clearance = −0.59 (age) + 3.96

where the clearance and constant term are both in mL min^−1^ kg^−1^, age is in years, and the coefficient of age has the unit mL min^−1^ kg^−1^ y^−1^.

The relative paucity of pharmacokinetic and pharmacodynamic drug data for children has led to the development of methods such as allometric scaling and mathematical modelling to estimate paediatric doses of drugs which undergo metabolism by CYP isozymes.^21^^,^^22^ The mathematical models are often based on data relating to physiological hepatic development and serum protein levels.^22^ Such is the case for the recent model by Suzuki and colleagues, which was developed for use in children up to the age of 15 years; it does not directly include the age of the child but instead incorporates the body surface area and estimates the child to adult dose ratio, which in turn is equal to the child to adult clearance ratio.^22^ Its accuracy has been tested for caffeine and theophylline, using population pharmacokinetic data.^22^

We hope that our current findings may be helpful in furthering the clinical use of the salivary caffeine clearance as a non-invasive test of hepatic function in children, as well as allowing refinement of mathematical modelling of paediatric dosing of CYP isozyme-metabolised drugs.

## 5 Conclusion

Our findings suggest that there is no need for stratification by sex when considering childhood salivary caffeine clearance or when conducting clinical or pharmacokinetic investigations in children of CYP1A2 and xanthine oxidase substrates. Furthermore, childhood salivary caffeine clearance is negatively correlated with age.

## Declaration of competing interest

None.
